# Effect of urea-extracted sericin on melanogenesis: potential applications in post-inflammatory hyperpigmentation

**DOI:** 10.1186/s40659-018-0204-5

**Published:** 2018-11-29

**Authors:** Pornanong Aramwit, Natthanej Luplertlop, Tapanee Kanjanapruthipong, Sumate Ampawong

**Affiliations:** 1 Bioactive Resources for Innovative Clinical Applications Research Unit and Department of Pharmacy Practice, Faculty of Pharmaceutical Sciences, Chulalongkorn University, PhayaThai Road, Phatumwan, Bangkok, 10330 Thailand; 2 Department of Microbiology and Immunology, Faculty of Tropical Medicine, Mahidol University, Ratchawithi Road, Ratchathewi, Bangkok, 10400 Thailand; 3 Department of Tropical Pathology, Faculty of Tropical Medicine, Mahidol University, Ratchawithi Road, Ratchathewi, Bangkok, 10400 Thailand

**Keywords:** Antityrosinase, Melanogenesis, MITF, Post inflammatory hyperpigmentation, Sericin

## Abstract

**Background:**

Hyperpigmentation disorders such as post-inflammatory hyperpigmentation are major concerns not only in light-skinned people but also in Asian populations with darker skin. The anti-tyrosinase and immunomodulatory effects of sericin have been known for decades. However, the therapeutic effects of sericin on hyperpigmentation disorders have not been well documented.

**Methods:**

In this study, we used an in vitro model to study the anti-tyrosinase, tolerogenic, and anti-melanogenic effects of sericin on *Staphylococcus aureus* peptidoglycan (PEG)-stimulated melanocytes, dendritic cells (DCs), and artificial skin (MelanoDerm™). Enzyme-linked immunosorbent assay, conventional and immunolabeled electron microscopy, and histopathological studies were performed.

**Results:**

The results revealed that urea-extracted sericin has strong anti-tyrosinase properties as shown by a reduction of tyrosinase activity in melanin pigments both 48 h and 10 days after allergic induction with PEG. Anti-inflammatory cytokines including interleukin (IL)-4, IL-10, and transforming growth factor-β were upregulated upon sericin treatment (10, 20, and 50 µg/mL), whereas production of allergic chemokines, CCL8 and CCL18, by DCs was diminished 48 h after allergic induction with PEG. Moreover, sericin lowered the expression of micropthalmia-associated transcription factor (MITF), a marker of melanogenesis regulation, in melanocytes and keratinocytes, which contributed to the reduction of melanin size and the magnitude of melanin deposition. However, sericin had no effect on melanin transport between melanocytes and keratinocytes, as demonstrated by a high retention of cytoskeletal components.

**Conclusion:**

In summary, sericin suppresses melanogenesis by inhibition of tyrosinase activity, reduction of inflammation and allergy, and modulation of MITF function.

## Background

Sericin is a water-soluble protein that anchors fibroin molecules together to form silk cocoons. This kind of silk protein is synthesized by *Bombyx mori* (silkworm) in the silk production, which is not naturally presented in human. Recently, several studies have recognized the various biological activities of sericin; hence, sericin has become a promising material in biomedical applications, particularly for promoting collagen formation in wound healing [1–5] and accelerating osteogenesis in bone repair processes [6, 7]. Sericin has been recognized to have immunomodulatory activities, showing anti-proliferative activity toward stimulated peripheral blood mononuclear cells and the ability to reduce production of pro-inflammatory cytokines including interleukin (IL)-1β, tumor necrosis factor (TNF)-α, and nitric oxide [8–11].

Hyperpigmentation disorders, particularly post-inflammatory hyperpigmentation (PIH) and melasma, have become important health concerns for which therapeutic approaches are lacking. PIH is more common in individuals with darker skin types (III–V), resulting in prevalence in some Asian populations up to 30–40% [12, 13]. The International Commission on Non-Ionizing Radiation Protection has stated that individuals with skin types I and II (light skin) are not uniquely at risk of melanoma and other adverse health effects due to UV rays. Additionally, individuals under the age of 18 are also at risk if they (i) have a large number of moles and/or a tendency to freckle, (ii) have a history of sunburn, (iii) wear cosmetics that enhance UV sensitivity, or (iv) take certain types of medication [14]. Several products have been investigated for their efficacy in preventing UV damage, but most of these are synthetic materials that pose risks of adverse reactions. Natural products with protective effects against UV rays are still under investigation. Recently, sericin has shown several biological effects, including UV-protective activity. Nevertheless, the immunomodulatory effects of sericin on melanocytes and dendritic cells (DCs), in connection with the localized epidermal immunology affecting hyperpigmentation disorders, are not well understood.

The most common target for inhibitors of hyperpigmentation is tyrosinase, the key regulator of melanin production [15] in association with micropthalmia-associated transcription factor (MITF), the master regulator for melanogenesis and melanocyte function [16, 17]. Therefore, many therapeutic products for PIH contain various active ingredients aimed at reducing melanin production and distribution. Importantly, sericin has been reported to be an inhibitor of tyrosinase activity [11, 18, 19]. However, little is known about the anti-melanogenic properties of sericin, including its effects on melanogenesis and melanin transportation as well as its pre-clinical efficacy.

In this study, we used an in vitro model of hyperpigmentation to characterize (i) the effect of sericin on production of the chemokines CCL8, CCL18, and CXCL10 by DCs after allergic stimulation with peptidoglycan (PEG), (ii) the tolerogenic effect of sericin on melanocytes, DCs, and artificial skin (MelanoDerm™), as shown by the expression of IL-4, IL-10, and transforming growth factor (TGF)-β, (iii) the anti-melanogenic properties of sericin on melanocytes and artificial skin, as shown by MITF expression, and (iv) the anti-tyrosinase effect of sericin on melanocytes and artificial skin. Our results expand the understanding of the immunomodulatory functions of silk sericin that underlie its effects in hyperpigmentation disorders. These findings may lead to improved therapeutic approaches.

## Materials and methods

### Ethics statement

Human participants or tissue samples were not involved in this study unless Buffy-coat samples, which purchased from Nation Blood Centre, Thai Red Cross Society, Thailand with anonymous achievement. However, following the guidelines of the Office of Human Research Protection (OHRP) and other national regulations, we have been obtained the permission. Therefore, this study was approved by the Ethics Committee of the Faculty of Tropical Medicine (FTM-EC), Mahidol University, with an exemption criterion (submitted Protocol Number TMEC 16-043).

### Sericin extraction

There are several methods for sericin extraction such as treatment with heat, acid, alkali, or urea. Previous studies have demonstrated that urea extraction of sericin yields the highest anti-tyrosinase activity. In this study, therefore, urea extraction of sericin was performed, as described by Aramwit [18]. Freshly-cut cocoon shells were soaked in 8 M urea for 30 min and then refluxed at 85 °C for 30 min. Insoluble residues were removed by centrifugation and filtration. The solution was thoroughly dialyzed in distilled water using a cellulose membrane for 3 days.

### Cells and culture methods

#### Melanocytes

The human melanoma cell line (ATCC^®^ CRL-1676) was cultured in Eagle’s Minimum Essential Medium supplemented with 10% (v/v) heat-inactivated fetal bovine serum and 1% (v/v) penicillin/streptomycin. Cells were grown at a density of 10^6^ cells/mL and maintained at 37 °C with 5% CO_2_ in a humidified incubator.

#### Dendritic cells (DCs)

DCs were kindly provided by Assoc. Prof. Dr. Natthanej Luplertlop, Department of Microbiology and Immunology, Faculty of Tropical Medicine, Mahidol University. DCs were isolated from human peripheral blood mononuclear cells by density centrifugation over Ficoll-Paque Plus (Sigma-Aldrich, Germany) [20]. Magnetic beads coated with antibodies (Abs; Miltenyi Biotec, France) were used to purify monocytes by negative selection. The monocytes were seeded at 10^6^ cells/mL in RPMI-1640 medium supplemented with 10% (v/v) fetal calf serum, 1% penicillin/streptomycin, 50 ng/mL recombinant human IL-4 (PeproTech, France), and 100 ng/mL recombinant human granulocyte–macrophage colony-stimulating factor (PeproTech, France) and cultured for 7 days at 37 °C with 5% CO_2_ in a humidified incubator.

#### Artificial skin (MelanoDerm™)

The 3D co-culture of keratinocytes and melanocytes, MelanoDerm™ (MatTek Corporation, USA), is a ready-to-use cell kit. Artificial skin was maintained in EPI-100-LLMM-AFAB-specific complete medium in 24-well plates. The medium was changed every other day without sub-culturing. Cells were grown and maintained at 37 °C with 5% CO_2_ in a humidified incubator.

### Experimental protocols

#### Anti-melanogenicity

To determine the anti-melanogenic properties of sericin, melanocytes and MelanoDerm™ were used. An allergic reaction in melanocytes was induced using 10 µg/mL PEG from *Staphylococcus aureus* (Sigma-Aldrich, Germany) for 24 h prior to sericin treatment to allow melanin production. Then, various concentrations of sericin (5, 10, and 20 µg/mL) were applied to the allergic melanocyte for 48 h. The melanocytes were trypsinized with 0.25% Trypsin–EDTA and washed in phosphate-buffered saline (PBS). The pellets were collected by centrifugation at 3500 rpm at 4 °C for 5 min and then fixed with 2.5% (v/v) glutaraldehyde in sucrose phosphate buffer (SPB) for 1 h. Fixed cells were washed in SPB and kept in 4 °C until the next steps.

To confirm the effect of sericin on melanin production by a more complex cell system, artificial skin was co-cultured with 10 µg/mL of *S. aureus* PEG and either sericin (5, 10, and 20 µg/mL) or culture medium as negative control for 10 days to allow melanin synthesis. During the experiment, all of the treatment and culture media were changed every 2 days. After 10 days, the artificial skin was separated into three parts and either (i) fixed in 10% neutral buffer formalin for histopathological studies, (ii) frozen at − 20 °C for the melanin assay, or (iii) fixed with 2.5% glutaraldehyde in SPB for electron microscopy.

#### Tolerogenicity

##### Chemokine production

To determine the effect of sericin on allergy-related chemokine production in DCs stimulated with *S. aureus* PEG, the production of CCL8, CCL18, and CXCL10 was assessed. CCL-8 and -18 are chemotactic factors attracting immune cells such as DCs, monocytes, macrophages, mast cells, eosinophils, and basophils, which are involved in allergic immune responses, particularly in atopic dermatitis [21]. CXCL10 is also responsible for T cell activation in various skin inflammatory diseases [22–24]. DCs were exposed to PEG with or without sericin treatment (1, 5, 10, 20, and 50 µg/mL) for 48 h. The dendritic cell pellet was trypsinized, centrifuged, collected, and fixed, as previously described, for electron microscopy studies. The supernatant was collected and frozen at − 20 °C until chemokines were measured using enzyme-linked immunosorbent assay kits (MyBiosource, USA).

##### Anti-inflammatory cytokine production

TGF-β, IL-4, and IL-10 are the major types of anti-inflammatory cytokines, which play roles in immune responses and enhance tolerogenicity. To determine the tolerogenic effect of sericin on allergic melanocytes, DCs, and MelanoDerm™, cytokine levels were evaluated using the immunogold labeling technique.

#### Anti-tyrosinase activity

To evaluate the tyrosinase antagonistic effect of sericin on melanocytes and artificial skin, tyrosinase expression was examined and compared between sericin-treated and non-treated groups using immunoelectron microscopy.

### Histopathology

#### Tissue processing

Fixed artificial skins were subjected to standard tissue processing: washed in tap water three times, dehydrated in grading ethanol, immersed in a paraffin series, embedded in pure paraffin, sectioned into 5-µm-thick sections, and stained with Hematoxylin and Eosin. The sections were examined by light microscopy focusing on melanin pigments.

#### Immunohistochemistry

Cytokeratin plays an important role in melanin transport from the melanocytic perinuclear region to the cell membrane and from melanocytes to neighboring keratinocytes [17]. Cytokeratin expression in artificial skin was characterized by immunohistochemical staining using a pan-cytokeratin antibody (AE1/AE3; Santa Cruz Biotechnology, USA). An antigenicity was retrieved using microwave-induced antigen retrieval in citrate buffer (pH 6.0). The non-specific binding and peroxidase blocking, HRP conjugated with secondary antibody, and chromogenic substrates were performed using the EnVision FLEX/HRP kit (DAKO, Denmark). Finally, the sections were counter-stained with Hematoxylin. Cytokeratin expression was examined under a light microscope. Measurements were performed using ImageJ program (NIH). The acquired images, containing at least 10 areas per group, were examined as a percentage area of expression per high power field (400×) [25–27].

### Electron microscopy

#### Conventional electron microscopy

Fixed melanocytes, DCs, and artificial skins were secondarily fixed in 0.1 M SPB containing 1% osmium tetroxide for 1 h. The specimens were dehydrated in an ethanol series, immersed in a series of LR white resin (EMS^®^, USA), embedded in pure resin, sectioned into 90–100-nm-thick sections, and stained with uranyl acetate and lead citrate. The ultrastructure of melanocytes and keratinocytes was evaluated under a transmission electron microscope (TEM; HT7700, HITACHI, Japan).

#### Immunogold labeling electron microscopy

To assess the effects of sericin, particularly its melanogenic, tolerogenic, and anti-tyrosinase effects, rabbit antibodies against MITF, IL-4, IL-10, TGF-β, and tyrosinase were used in the immunogold labeling technique. Melanocytes, DCs, and artificial skins sections were aldehyde-blocked in PBS containing 50 mM glycine (pH 7.4). The sections were blocked in PBS (pH 7.4) containing 5% (w/v) bovine serum albumin (BSA; EMS, USA). The sections were then washed in incubation buffer (0.1% BSA in PBS, pH 7.4) and incubated with primary antibodies for 1 h. The secondary antibody, goat anti-rabbit IgG conjugated with 10 nm gold (Sigma-Aldrich, Germany), was applied to the sections for 1 h. After rigorous washing with incubation buffer and distilled water, the sections were incubated with the silver enhancement kit (EMS, USA) and stained with uranyl acetate and lead citrate to increase the contrast. To quantify the expression level, the number of immune-positive gold particles was counted focusing on specific areas of interest depending on the cell type. At least 10 fields per group were examined under TEM.

### Melanin assay

Melanin deposition in artificial skin was evaluated using air-drying assay. Frozen artificial skins were dissolved in 7.5 N NaOH for 3 h in a 60 °C oven. The solutions were measured in a micro-plate reader at absorbance ranges between 380 and 440 nm. The results were compared for MelanoDerm™ treated with or without sericin.

### Melanin size measurement

The sizes of melanin pigments in the melanocytes after treatment with or without sericin were determined using ImageJ program. The ultrastructural images were acquired, focusing on the melanin pigments. Fifty melanin pigments were measured and the size in µm^2^ was determined by drawing a line over the area of interest under area measurement mode.

### Statistical analyses

Statistical analyses were conducted using GraphPad Prism^®^ version 5. The student’s t-test or Dunnett’s test were used to assess differences between group means of quantitative parameter. Significance level was considered at p < 0.05.

## Results

### Anti-melanogenic and anti-tyrosinase effects of sericin

#### Ultrastructural visualization of melanin transport in artificial skin

**Fig. 1:**
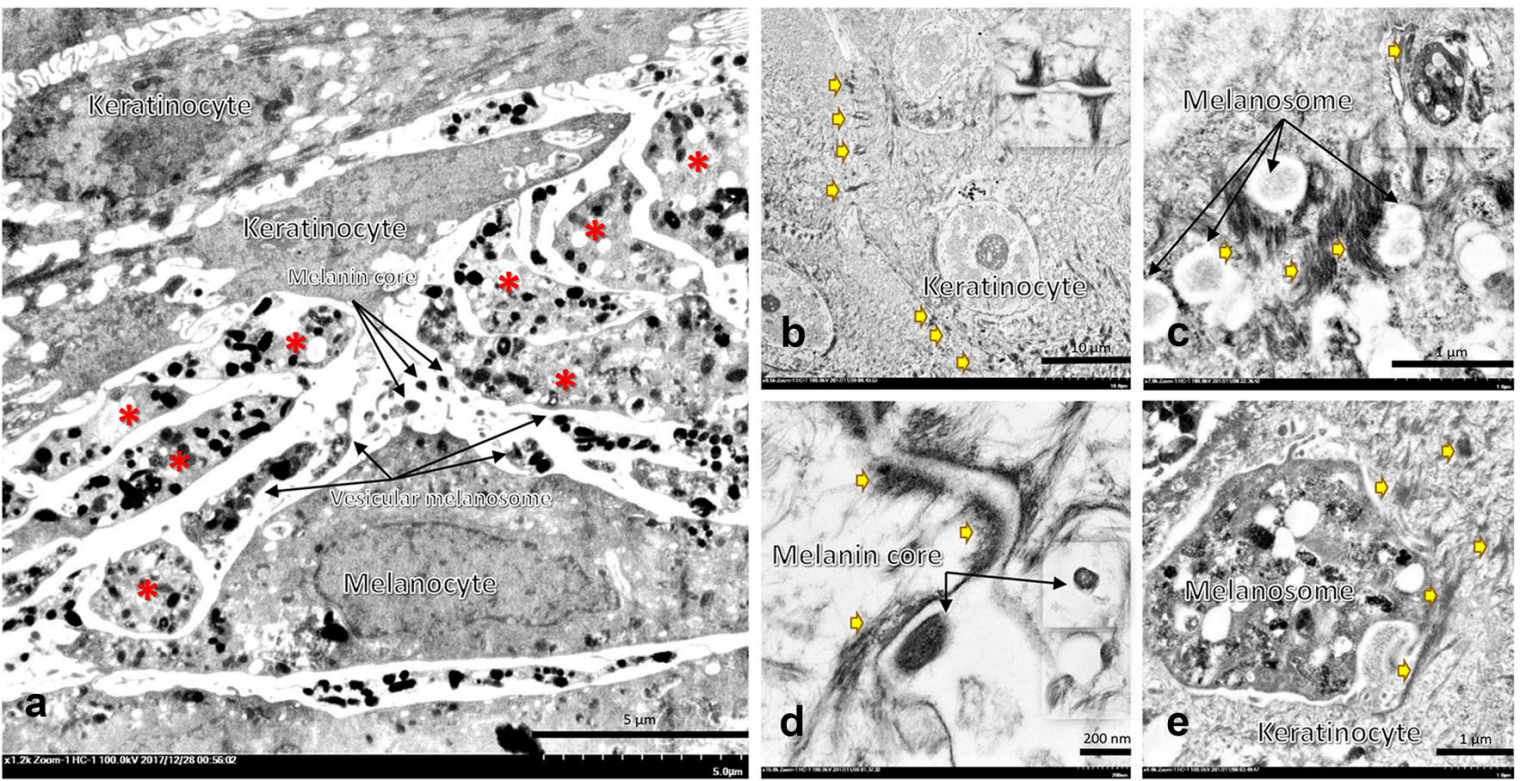
Electron micrographs of artificial skin 10 days after treatment; At the artificial skin bottom layer, melanocytes extended multiple dendritic extensions (**a**: *) acting as a pipe for transport of mature melanosomes to neighboring keratinocytes, which bore cell-to-cell connections via desmosomes (**b**: arrow and inset picture and **d**: arrow). Immature melanin is transferred from the perinuclear region to dendritic tips, a maturation site, via the cytoskeletal system (**c**, **e**: thick arrow). Four mechanisms of melanin transport have been postulated: (i) direct fusion of melanocytes and keratinocytes and (ii–iv) keratinocyte phagocytosis (**c**: inset picture, **e**) of (ii) membrane-bound vesiculate melanosomes (**a**) (iii) exocytosis of melanin cores from dendritic tips (**a**, **d**), and (iv) dendritic tips

Electron microscopy of artificial skin revealed that melanocytes and keratinocytes are the major cell types in MelanoDerm™ producing melanin and keratin, respectively. The fine morphological appearance of melanin synthesis and transport are shown in Fig. 1.

#### Anti-tyrosinase effect of sericin

**Fig. 2:**
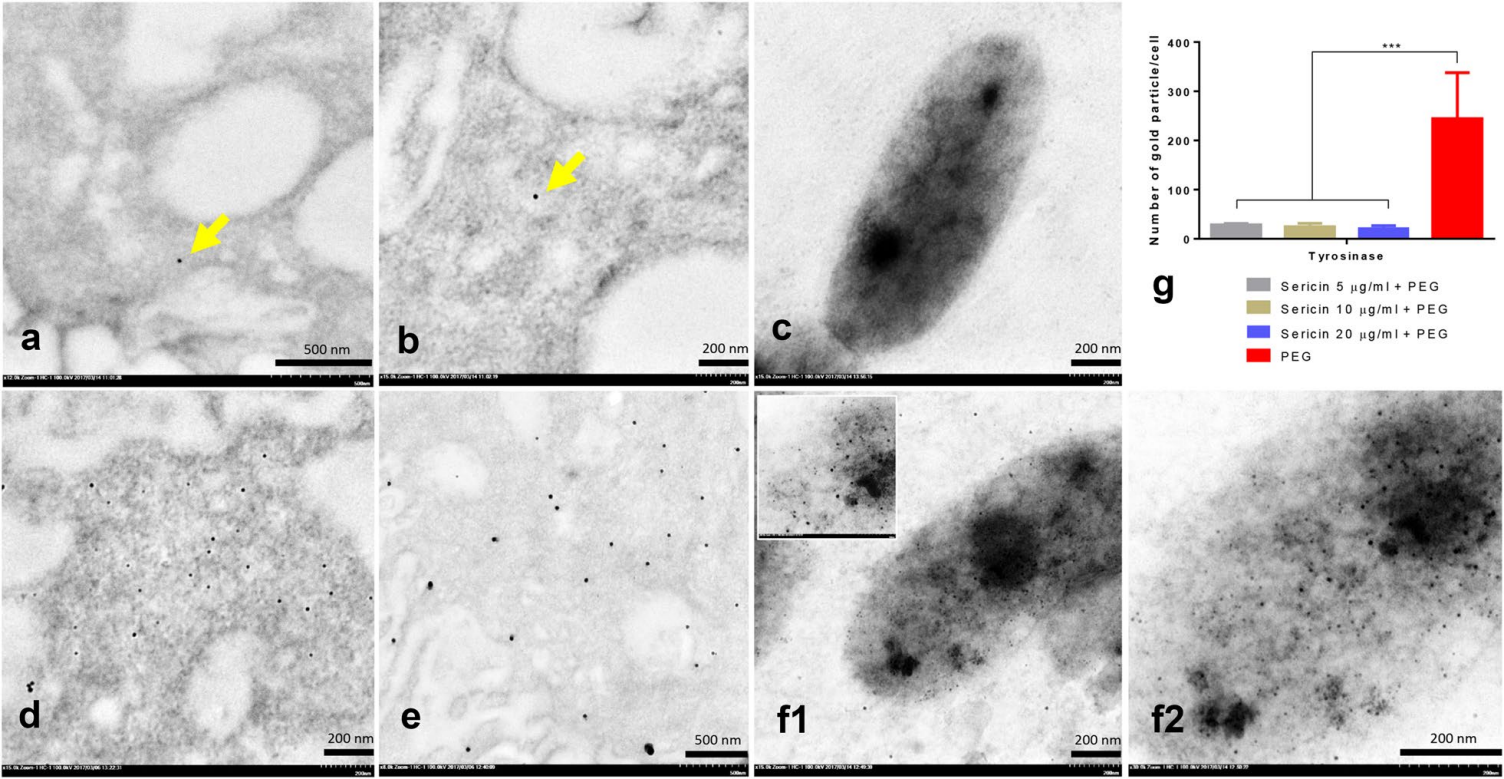
Tyrosinase gold labeling on melanocytes; Immunogold labeling electron microscopy showing the 10 nm diameter gold particles conjugated to tyrosinase molecules on melanocytes both in the cytoplasm (arrow in **a**, **b** and **d**, **e**) and melanosomes (**c** and **f**-**1**, low magnification, -**2**, higher magnification). The quantity of tyrosinase between sericin-treated (**a**–**c**) and non-treated (PEG) (**d**–**f**) groups was compared via the numbers of tyrosinase-positive fields in melanocytic cells as shown in bar graph (**g**)

**Fig. 3:**
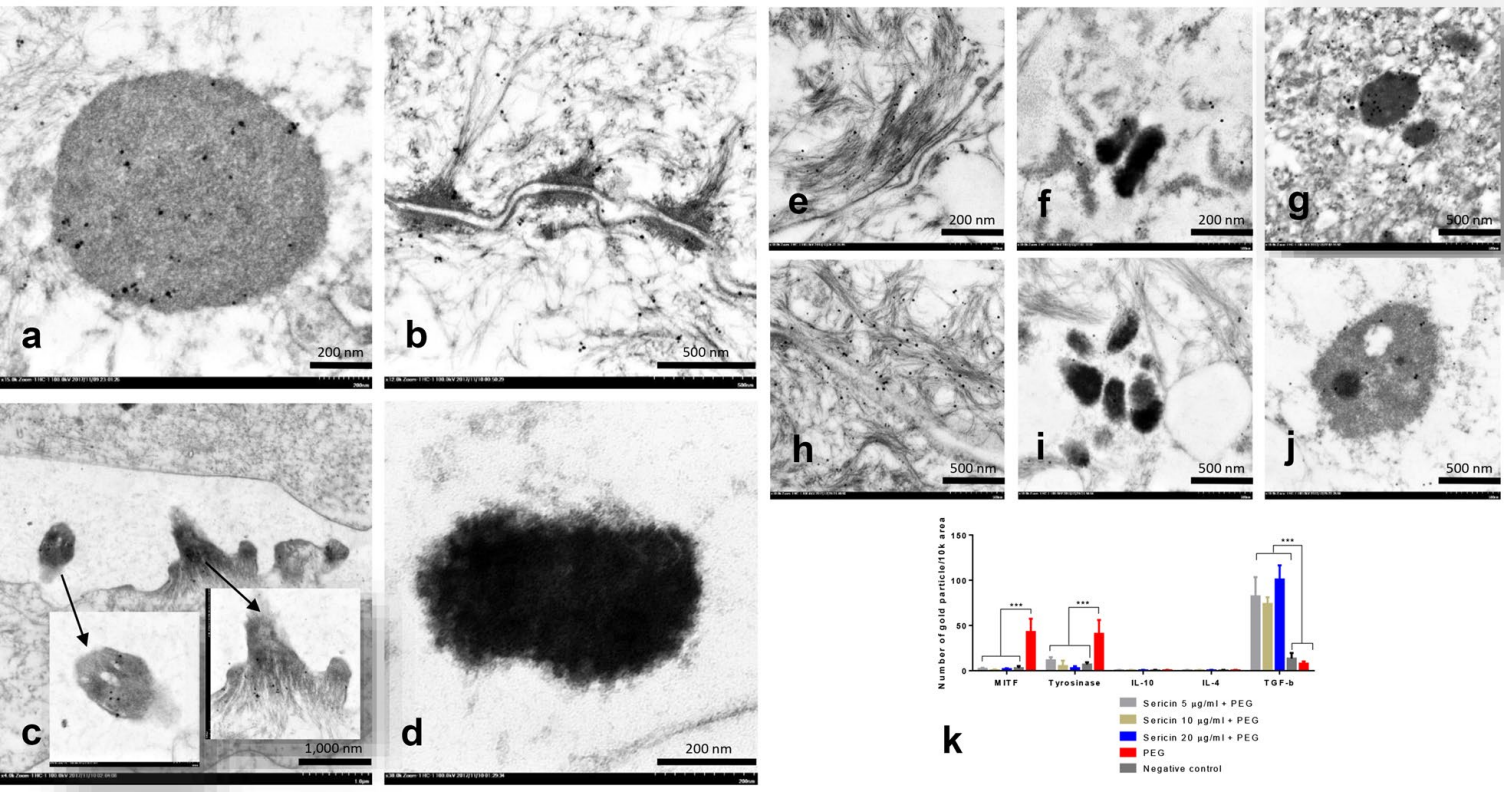
Immunogold labeling of tyrosinase, MITF, IL-4, IL-10, and TGF-β in artificial skin 10 days after treatment; Tyrosinase was thoroughly labeled in melanocytes and keratinocytes, especially on immature melanosomes (**a**), keratinocytic desmosomes and the cytoskeletal system (**b**), and cortical actin in the melanocyte dendritic tip (**c**), but not in mature melanin (**d**). Like tyrosinase, TGF-β (**e**–**g**) and MITF (**h**–**j**) were labeled on the cytoskeletal system and melanin was labeled in the melanocyte and keratinocyte. All sericin doses downregulated MITF and tyrosinase expression, upregulated TGF-β expression but did not affect expression of IL-4 and IL-10 (**k**)

Gold labeling of tyrosinase showed that PEG enhanced tyrosinase production within 48 h of allergic induction in melanocytes. Interestingly, all sericin doses significantly inhibited tyrosinase production on melanin pigments when compared with non-treated melanocytes (Fig. 2). Therefore, sericin has anti-tyrosinase effects during allergic reactions. We also observed that tyrosinase was labeled on immature melanin, keratinocytic desmosomes, melanocytic and keratinocytic cytoskeletal systems, and dendritic tips of the melanocytes (Fig. 3a–d). In addition, 10 days after treatment with all sericin doses, tyrosinase expression in artificial skin was significantly reduced (Fig. 3k). These results reflected the role of tyrosinase in melanogenesis and melanin transport between melanocytes and keratinocytes.

#### Effects of sericin on MITF expression

**Fig. 4:**
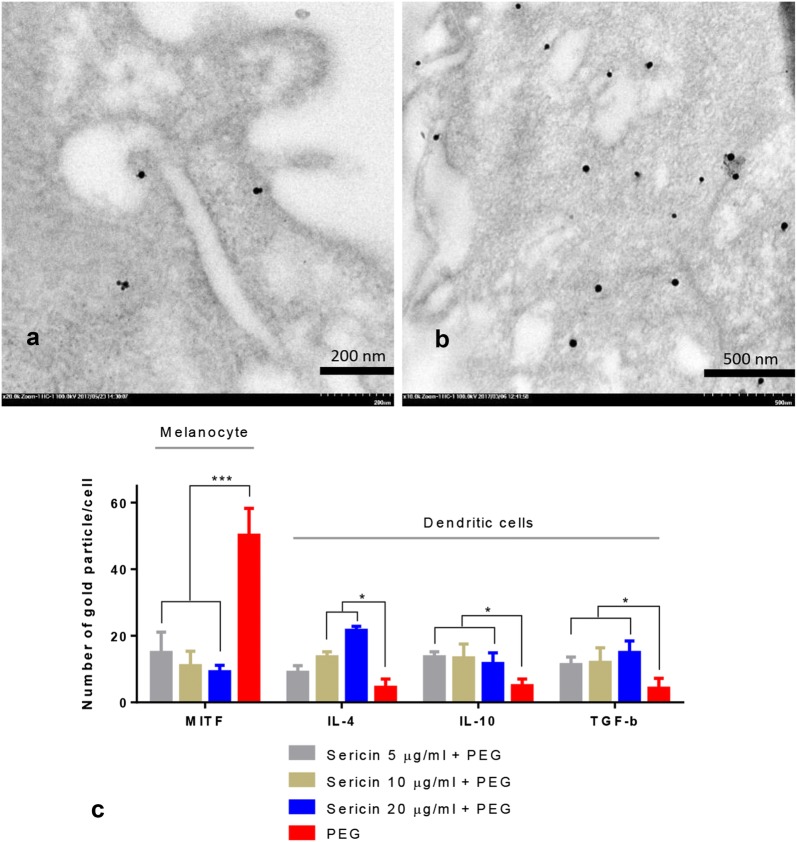
MITF, IL-4, IL-10, and TGF-β gold labeling on melanocytes and DCs 48 h after allergic induction; Electron micrograph of MITF immunogold labeling on melanocytes with (**a**) or without (**b**) sericin treatment indicated significant downregulation of MITF by all sericin doses when compared with non-treated cells as presented in bar graph (**c**). Conversely, expression of IL-4, IL-10 and TGF-β on DCs were significantly upregulated by all sericin doses (**c**). The results revealed that sericin has anti-melanogenic and immunomodulatory effects on epidermal cells during the 48 h after allergic stimulation

Both sericin-treated melanocytes and artificial skin had significantly lower MITF expression than non-treated groups (Figs. 3h–k and 4). Labeling of MITF and tyrosinase seemed to be closely related, as shown by their similar expression patterns in the cytoskeletal system and on immature melanin pigments.

#### Melanin levels in artificial skin are affected by sericin

**Fig. 5:**
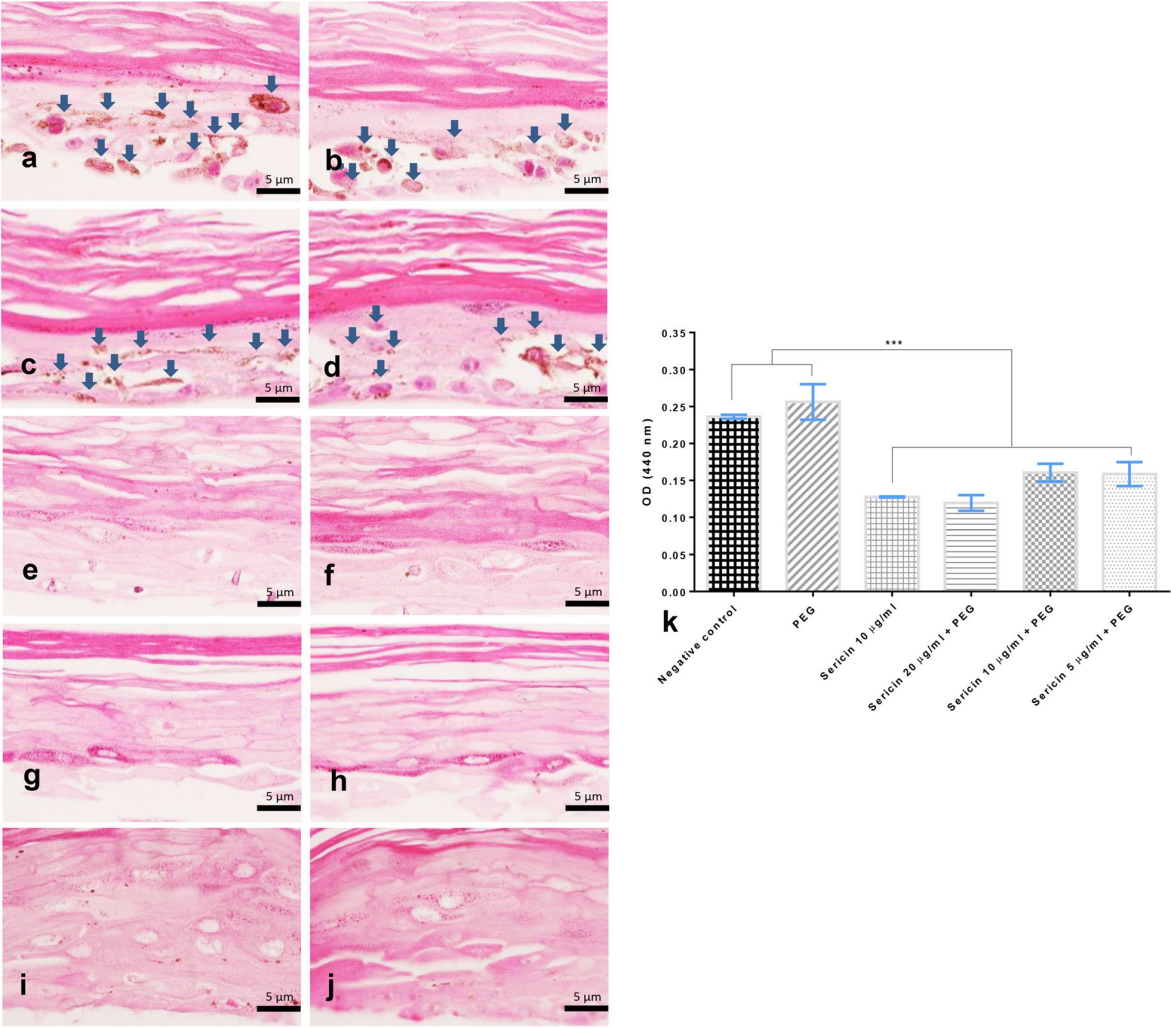
Melanin deposition in artificial skin 10 days after treatment; Histopathological appearance of the artificial skin indicating the epidermal layer (stratum corneum, granulosum, spinosum, and stratum basale) which is mainly composed of keratinization, keratinocytes and melanocytes with melanin (golden brown pigment) deposition (**a**–**d**; arrow). The melanin deposition level in the artificial skin both treated with sericin (**e**–**f**, **g**–**h**, and **i**–**j**; 5, 10, and 20 µg/mL, respectively) and untreated (**a**, **b** non-allergic and **c**, **d** allergic inductions, respectively) was compared in the bar graph (**k**)

Histopathological studies demonstrated that 10 days after treatment with sericin, melanin levels in non-treated skin (Fig. 5a, b) and negative control group skin (Fig. 5c, d) were significantly higher than those in sericin-treated skin (Fig. 5e–j), as shown in Fig. 5k. These results revealed that sericin has anti-melanogenic effects on melanocytic cells.

#### Melanin size

**Fig. 6:**
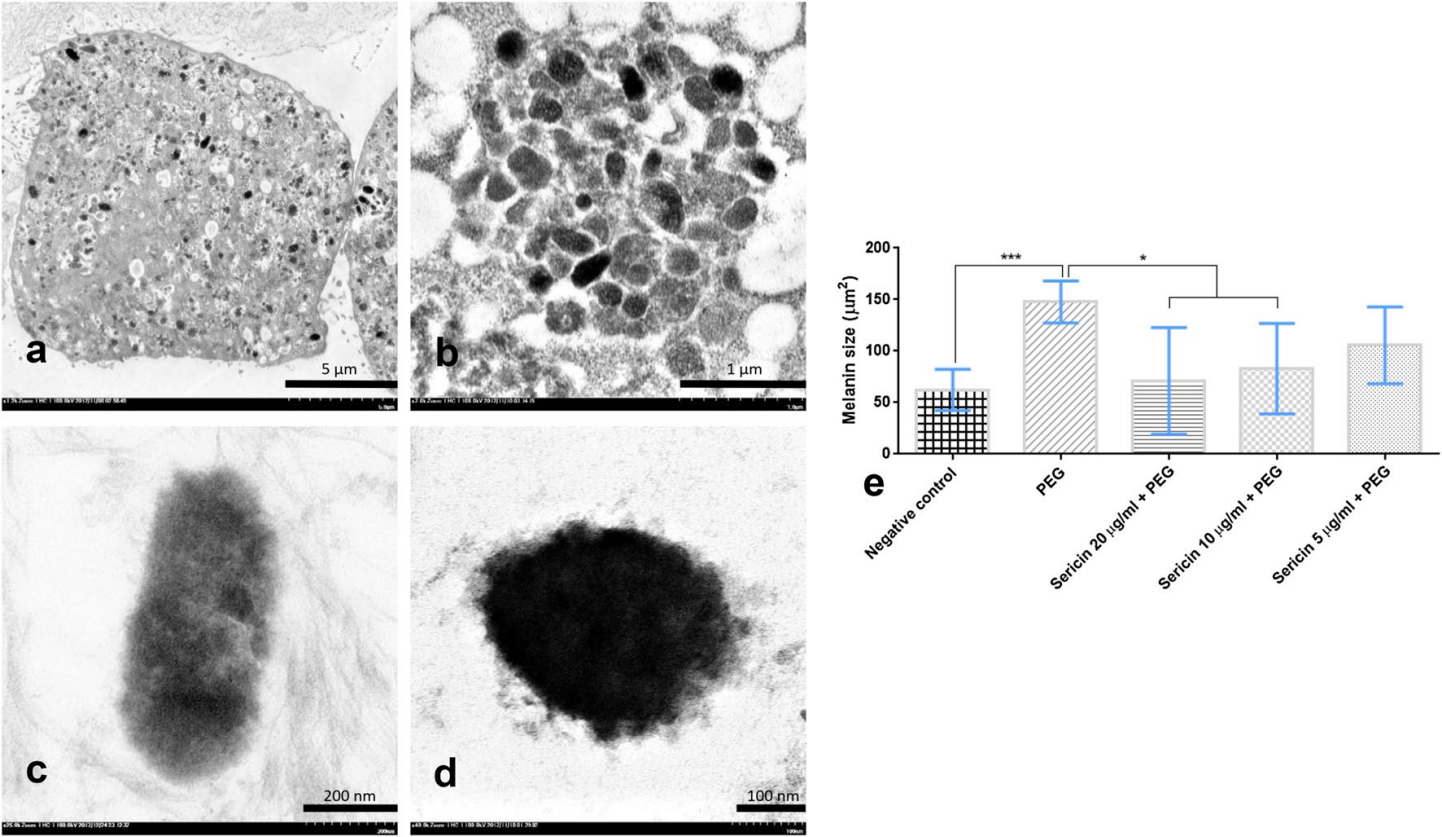
Melanin pigment size among sericin-treated groups compared with negative control and non-treated groups; Melanocyte laden melanin pigment (**a**) showed a number of melanin depositions in its cytoplasm with difference stages (**b**): immature (**c**) or mature (**d**) melanin pigments. Size comparison of melanin pigments is shown in the bar graph (**e**)

The size of melanin pigments is closely related to melanogenesis. Sericin treatment of melanocytes (10 and 20 µg/mL doses) significantly reduced the magnitude of melanin pigmentation compared with non-treated and negative control groups (Fig. 6). Unlike the higher dosages, 5 µg/mL sericin was insufficient to decrease the size of melanin pigments.

### Sericin reduces cytokeratin expression in artificial skin

**Fig. 7:**
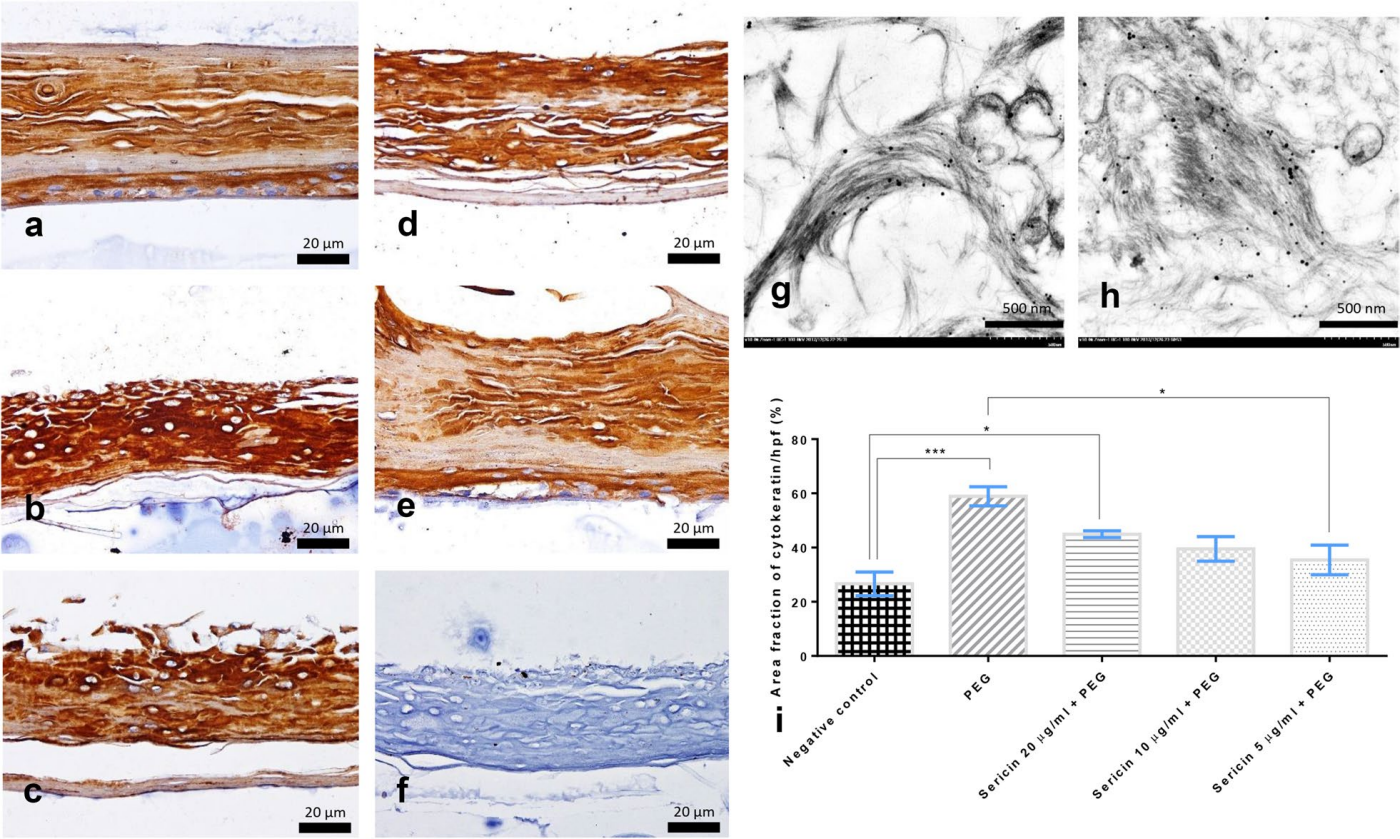
Cytokeratin expression in artificial skin 10 days after treatment; Immunohistochemical staining of cytokeratin indicating the distribution of keratin expression in keratinocytes and melanocytes from the indicated experimental group; negative control (**a**), non-treated group (**b**), sericin-treated with doses of 5 (**c**), 10 (**d**), and 20 (**e**) μg/mL comparing to non-immunostained skin (**f**). Tyrosinase was mainly labeled on the cytoskeletal components that are characterized by numerous of gold particles (**g**, **h**). Cytokeratin level was compared as shown in the bar graph (**i**)

Immunohistochemical studies of cytokeratin demonstrated that significantly lower expression was observed in the negative control group (Fig. 7a) than in untreated PEG-induced skin (Fig. 7b) and in 20 µg/mL of sericin-treated skin (Fig. 7c). However, only low sericin doses (5 µg/mL) significantly decreased cytokeratin expression (Fig. 7e). Interestingly, tyrosinase was highly labeled on the cytoskeletal components in the cytoplasm of melanocytes and keratinocytes (Fig. 7g, h). Cytokeratin expression (Fig. 7a–e) seemed to be associated with tyrosinase levels, potentially reflecting a joint role in melanin transportation.

### Tolerogenic effects of sericin

#### Effects on anti-inflammatory cytokines

Anti-inflammatory cytokines play an important role in decreasing inflammation and allergic reactions in the skin, leading to the alleviation of PIH. DCs are the major production site of the mediators on superficial innate immune responses. Immunogold labeling electron microscopy showed that 48 h after allergic induction of DCs, all sericin doses significantly increased the levels of IL-4, IL-10, and TGF-β when compared with non-treated DCs (Fig. 4c). By contrast, in the artificial skin model, all groups had similar low levels of IL-4 and IL-10 production 10 days after treatment. This may relate to the cellular components of MelanoDerm™, which lacks DCs, a major immunoregulatory cell type in the skin. However, treatment of artificial skin with all sericin doses resulted in significantly higher TGF-β levels than those in non-treated and negative control groups (Fig. 3k). Therefore, sericin affected production of some anti-inflammatory cytokines to alleviate skin inflammation and allergy.

#### Effects on chemokines

**Fig. 8:**
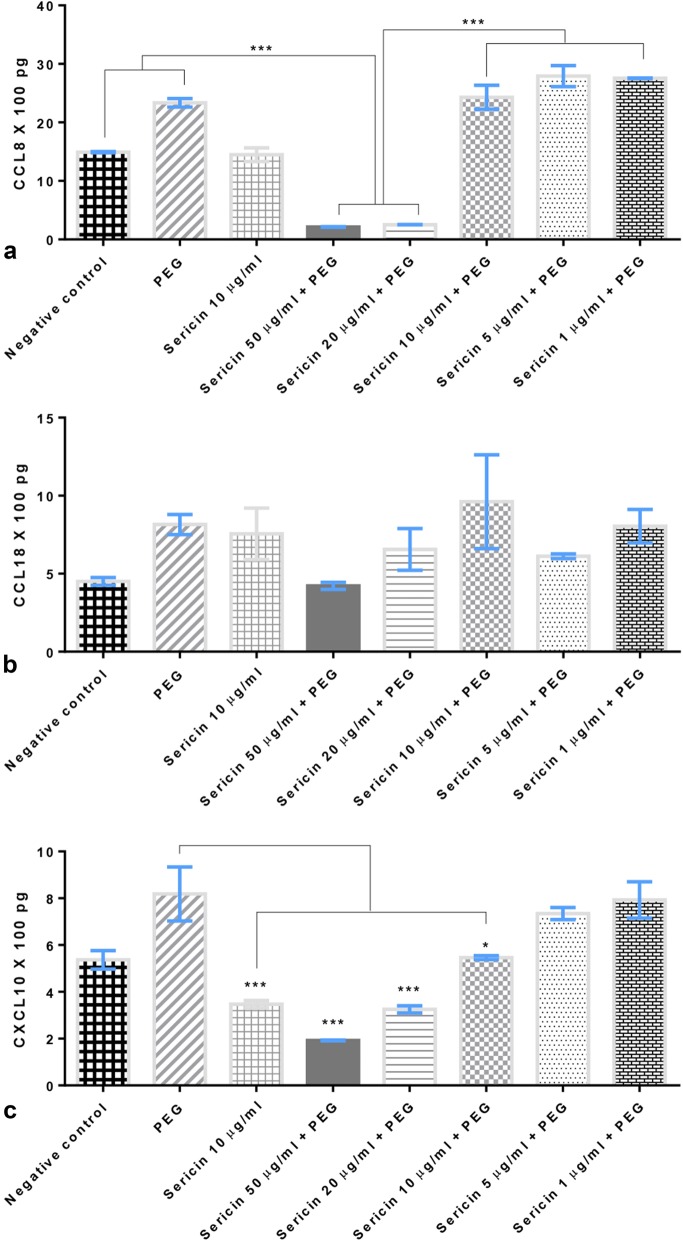
Chemokine production by dendritic cells 10 days after treatment; CCL8 (**a**), CCL18 (**b**) and CXCL10 (**c**) productions from allergic-induced DCs with or without sericin treatment

Chemokine-induced allergy is an important synergistic factor enhancing melanogenesis. We assessed the levels of some chemokines produced from DCs 48 h after allergic induction. Sericin (20 and 50 µg/mL doses) significantly reduced CCL8 and CXCL10 levels compared with non-treated DCs (Fig. 8a, c). However, CCL18 (Fig. 8b) levels were not reduced by sericin treatment.

## Discussion

Hyperpigmentation disorders, particularly PIH, a common sequelae of inflammatory dermatoses, and melasma, acquired hypermelanosis of sun-exposed skin, tend to affect darker skinned patients with higher frequency and severity worldwide including African Americans, Hispanics/Latinos, Asians, Indians, Native Americans, Pacific Islanders, and Middle Eastern descent [12, 28]. First-line therapy usually aims to decrease melanin synthesis using topical depigmentation agents, which compose of tyrosinase inhibitor, photoprotector and whitener. Regarding to the desired properties of sericin based on our present results, sericin has a potential capability for PIH and melasma alleviations and therapeutic approaches.

In this study, we used in vitro models of allergy induction both in single cells (melanocytes and DCs) and more complex cell systems (artificial skin; MelanoDerm™) to examine the anti-tyrosinase, tolerogenic, and anti-melanogenic properties of urea-extracted sericin. The results indicated that sericin plays a role in suppressing melanogenesis via its tyrosinase antagonism and anti-inflammatory properties, resulting in decreased melanin deposition and pigment size in artificial skin (Figs. 2, 3, 4, 5, 6 and 8). However, sericin did not inhibit melanin transport between melanocytes and keratinocytes, as shown by the unperturbed cytoskeletal components (Fig. 7).

The skin, an important barrier from physical and microbiological injuries, comprises multiple cell types. Melanocytes and resident DCs are located in the epidermis and function as important modulators of mucosal innate immune responses [29–31]. In the skin, chemokines are secreted by both resident cells (particularly melanocytes and DCs) and infiltrating cells, leading to induction and maintenance of inflammation [32]. Melanocytes are also responsible for synthesis and transport of melanin pigments to keratinocytes through several postulated mechanisms [17]. Some of the well-described mechanisms are demonstrated in this study, as shown in Fig. 1. Alterations of melanocyte function leads to several skin manifestations such as rash, hyper- or de-pigmentation disorders, epidermolysis, and psoriasis-like lesions [29].

PIH, a hyperpigmentation disorder, occurs subsequent to skin inflammation or injury (burns, acne, psoriasis, and friction) and leads to dark plaques on the skin. Although PIH can resolve spontaneously, the process takes months or more. Subsequent to skin injuries, cytokines and inflammatory mediators (particularly leukotrienes, prostaglandin E2, thromboxane-2, IL-1, IL-6, and TNF-α) can stimulate melanocyte activity and promote melanin production [33]. Our study revealed that sericin decreased production of allergic chemokines, CCL8 and CCL18, and increased production of anti-inflammatory cytokines, IL-4, IL-10, and TGF-β. These effects served to preserve a tolerogenic state, thus modulating the degree of inflammation and resulting in lower melanin production. Anti-inflammatory activity of sericin has also been reported by our colleagues. Sericin reduces inflammatory responses in carrageenan-induced allergy [34] and suppresses COX-2 enzyme activity and nitric oxide production [10].

Unlike PIH, the association between melanoma and superficial innate immune responses is not well understood. Experiments using an interesting animal model, the silky fowl (*Gallus gallus domesticus* Brisson), have suggested that hyperpigmentation may be related to inhibition of immune development and particularly to the degeneration of lymphoid organs [35]. The interaction between tyrosinase and MITF and its effect on melanogenesis has been well studied [17, 36, 37]. Melanogenic signaling requires *PAK1* (oncogenic aging kinase) and MITF for tyrosinase expression and a related protein product of *TRP* that is essential for melanin biosynthesis from tyrosine. Tyrosine is hydroxylated by tyrosinase to yield 3,4 dihydroxyphenylalanine (DOPA), DOPA quinine, and finally melanin pigment. Blocking any step in the melanin biosynthesis pathway is a useful step in investigating new therapeutic drugs for melanogenesis inhibition.

Recently, tyrosinase inhibition has been shown to be a desirable strategy to alleviate hyperpigmentation in the skin. Our previous study showed that like other natural products [38, 39], sericin exhibits anti-tyrosinase activity [18]. Different extraction methods may affect the conformation and proportion of amino acids in sericin, and in our study, urea extraction of sericin yielded higher anti-tyrosinase activity than acidic, basic, and heat extractions. Apart from serine, arginine and valine also enhance the anti-tyrosinase activity of sericin because they have high tyrosinase-binding and -inhibiting activities [40]. In addition, flavonoids and carotinoids from silk cocoon pigment synergistically affect the anti-tyrosinase property of sericin. Interestingly, branched-chain amino acids such as isoleucine, leucine, and valine [41] and dipeptides such as proline–serine and valine–serine [42] have anti-melanogenic properties. Although valine slightly inhibits tyrosinase activity, a complex of BCAAs impedes melanin production without affecting anti-tyrosinase activity. Additionally, dipeptides suppress melanogenesis through a mechanism related to ERK phosphorylation, leading to downregulation of MITF and tyrosinase. Therefore, the anti-melanogenic and anti-tyrosinase properties of sericin may be strongly related to its peptide components and their proportions. Urea-extracted sericin may be composed of a more complete amino acid sequence than that extracted using other methods, which is reflected by its activity. However, these mechanisms are complex and need to be further studied.

Other therapeutic agents for PIH use distinct mechanisms to inhibit melanogenesis. Hydroquinone and azelaic acid interfere with tyrosinase, whereas mequinol acts as a competitive substrate of tyrosinase [43]. Retinoids increase the penetration of other medications through the skin barrier by causing skin irritation and inducing the apoptosis of mature melanocytes [44]. Ascorbic acid suppresses melanin production by reducing the formation of quinones, creating a barrier in melanogenesis [45].

## Conclusions

In conclusion, our study demonstrated that sericin has dose-dependent anti-tyrosinase, anti-inflammatory, and anti-melanogenic effects on melanocytes, DCs, and artificial skins. These effects occurred through sericin’s inhibition of tyrosinase, allergic cytokine production, and MITF without inhibitory effects on melanin transport. These properties may be related to the sericin extraction methods and the integrity of its amino acid sequence. Sericin is a promising natural candidate for alleviating PIH and/or related hyperpigmentation disorders.

## Abbreviations

PEG: peptidoglycan
DCs: dendritic cells
IL: interleukin
TGF: transforming growth factor
MITF: micropthalmia-associated transcription factor
TNF: tumor necrosis factor
PIH: post-inflammatory hyperpigmentation
OHRP: Office of Human Research Protection
FTM-EC: Ethics Committee of the Faculty of Tropical Medicine
PBS: phosphate-buffered saline
SPB: sucrose phosphate buffer
DOPA: 3,4 dihydroxyphenylalanine
TRF: Thailand Research Fund
OHEC: Higher Education Commission
